# Immune Signatures Combined With BRCA1-Associated Protein 1 Mutations Predict Prognosis and Immunotherapy Efficacy in Clear Cell Renal Cell Carcinoma

**DOI:** 10.3389/fcell.2021.747985

**Published:** 2021-10-18

**Authors:** Ze Gao, Junxiu Chen, Yiran Tao, Qiong Wang, Shirong Peng, Shunli Yu, Jianwen Zeng, Kaiwen Li, Zhongqiu Xie, Hai Huang

**Affiliations:** ^1^Department of Urology, Sun Yat-sen Memorial Hospital, Sun Yat-sen University, Guangzhou, China; ^2^Guangdong Provincial Key Laboratory of Malignant Tumor Epigenetics and Gene Regulation, Sun Yat-sen Memorial Hospital, Sun Yat-sen University, Guangzhou, China; ^3^Department of Urology, The Sixth Affiliated Hospital, Sun Yat-sen University, Guangzhou, China; ^4^Department of Pathology, School of Medicine, University of Virginia, Charlottesville, VA, United States; ^5^Department of Urology, Qingyuan People’s Hospital, The Sixth Affiliated Hospital of Guangzhou Medical University, Qingyuan, China

**Keywords:** immunotherapy efficacy, clear cell renal cell carcinoma, prognosis carcinoma, immune signature, BAP1 mutation

## Abstract

Immunotherapy is gradually emerging in the field of tumor treatment. However, because of the complexity of the tumor microenvironment (TME), some patients cannot benefit from immunotherapy. Therefore, we comprehensively analyzed the TME and gene mutations of ccRCC to identify a comprehensive index that could more accurately guide the immunotherapy of patients with ccRCC. We divided ccRCC patients into two groups based on immune infiltration activity. Next, we investigated the differentially expressed genes (DEGs) and constructed a prognostic immune score using univariate Cox regression analysis, unsupervised cluster analysis, and principal component analysis (PCA) and validated its predictive power in both internal and total sets. Subsequently, the gene mutations in the groups were investigated, and patients suitable for immunotherapy were selected in combination with the immune score. The prognosis of the immune score-low group was significantly worse than that of the immune score-high group. The patients with BRCA1-associated protein 1 (BAP1) mutation had a poor prognosis. Thus, this study indicated that establishing an immune score model combined with BAP1 mutation can better predict the prognosis of patients, screen suitable ccRCC patients for immunotherapy, and select more appropriate drug combinations.

## Introduction

Kidney cancer was the 16th most common cancer in 2018, with 403,262 new cases and 175,098 deaths worldwide (Bray et al., 2018). Renal cell carcinoma comprises many histological subtypes, the most common of which is clear cell renal cell carcinoma (ccRCC), which accounts for 75% of all cases (Turajlic et al., 2018). Currently, the treatment of renal cancer is mainly surgical resection. However, approximately one-third of patients will relapse after surgery, and metastases are found in approximately 30% of patients at the time of initial diagnosis (Janzen et al., 2003). The advent of cytokine therapy, such as interleukin-2 (IL-2) and interferon alpha-2B (IFN-α), brought the earliest immunotherapy regimens (Margolin et al., 1989; Fisher et al., 2000). With the rise of targeted therapies for renal cancer, the effectiveness of vascular endothelial group factor (VEGF) and molecular target of rapamycin (mTOR) pathway inhibitors for metastatic renal cell carcinoma appears to limit the development of immunotherapy (Posadas et al., 2013). In recent years, the emergence of immune checkpoint blockade (ICB) therapy, which blocks programmed cell death protein 1 (PD-1) and programmed death-ligand 1 (PD-L1), has further advanced immunotherapy. Patients have benefited from treatment for lung cancer, Hodgkin’s lymphoma, and glioblastoma (Ansell et al., 2015; Forde et al., 2018; Cloughesy et al., 2019). Therefore, exploring the relevant indicators of immunotherapy effectiveness in ccRCC is necessary.

Immune cell infiltration and the tumor mutation burden (TMB) play key roles in the efficacy of tumor immunotherapy. Neoantigens are produced by a few somatic mutations in tumors and can be recognized by the immune system (Snyder et al., 2014). These mutations can be transcribed and translated and present in the MHC complex on the surface of tumor cells (Coulie et al., 2014). However, not all mutations can produce neoantigens, and not all neopeptides present on the cell surface can be recognized by T cells (Carreno et al., 2015; Snyder and Chan, 2015). Therefore, the search for effective mutations can further enhance the efficacy of immunotherapy. Tumors are complex new organisms that contain not only malignant tumor cells but also other types of cells. Among these cells, immune infiltrating cells play a central role in the immunotherapy response (Chen and Mellman, 2017). The levels of tumor-infiltrating CD8^+^ and CD4^+^ T cells are correlated with the immunotherapy response (Topalian et al., 2016). Cytotoxic CD8^+^ T cells are the main effectors of antitumor immunity and can specifically recognize and kill tumor cells carrying neoantigens (Chen and Mellman, 2013). However, not all immune cells can produce a positive immune response against tumors. In many cases, some immune cells are dysfunctional, leading to immunosuppression, supporting tumorigenesis and immune evasion, such as Treg cells (Finotello and Trajanoski, 2017). The molecules involved in Treg-mediated inhibition include IL-2, IL-10, TGF-β, IL-35, cytotoxic T lymphocyte-associated protein 4 (CTLA-4), glucocorticoid-induced TNF receptor (GITR), and cAMP (Tanaka and Sakaguchi, 2017). Therefore, quantifying the degree of immune cell infiltration in tumors, as well as the expression of immunosuppressive receptors and ligands, will help to select appropriate immunotherapeutic drugs.

In this study, to screen patients with ccRCC suitable for immune checkpoint inhibitor therapy, we assessed and quantified the level of immune infiltrating cells and screened differential genes to construct immune scores. We also explored the changes in tumor mutation burden in patients with different immune scores and combined tumor mutation burden to more accurately select immune checkpoint inhibitors (ICI) treatment patients in ccRCC.

## Materials and Methods

### Samples and Data Process

The RNA-seq data (level 3) of 530 ccRCC patients were obtained from The Cancer Genome Atlas (TCGA) database^1^. The masked somatic mutation data of 336 ccRCC patients were downloaded from the TCGA database. The R packages “limma” and “maftools” were used to process the RNA-seq and calculate the total number of somatic non-synonymous point mutations within each sample, respectively.

### Estimation of Immune Cell Type Fractions

To determine the cell composition in the tumor microenvironment, we used xCell and CIBERSORT to estimate immune cell types. CIBERSORT estimates immune cell subpopulations using RNA-Seq (Newman et al., 2015). It obtains aggregated high-dimensional data from tumor cell mixtures and infers cell composition based on the expression profile of purified white blood cell subpopulations. xCell uses a set of 10,808 genes to score and estimate the degree of infiltration of 64 cell types (Aran et al., 2017). It can further accurately distinguish the activation state of CD8^+^ T cells, a function that is impossible for CIBERSORT. To ensure the accuracy of the results, a *p*-value less than 0.05 was used as the criterion.

### Gene Set Enrichment

We used single sample gene set enrichment analysis (ssGSEA) to quantify the enrichment level of 29 immune features of each sample, including immune cell types, functions, and pathways (Barbie et al., 2009). According to the results, hierarchical cluster analysis was performed on all patients, who were divided into two groups. To identify the regulatory pathways with the largest differences between the two groups, the R package Pi containing 205,000 genes was used for gene set enrichment analysis, and 20,000 permutations were used (Fang et al., 2019). Additionally, we performed gene set variation analysis (GSVA) between the ISL group and ISH group using the GSVA package in R language.

### Constructed the Immune Score

To better measure the immune infiltration pattern and immune pathways of ccRCC, we constructed an immune score model using different immune infiltration and immune function groups. The construction process of the immune score was as follows:

First, all the samples were divided into two groups according to the activity, enrichment level, and function of immune infiltration cells. The differentially expressed genes (DEGs) were identified from the immune high group and immune low group with | log2FoldChange| > 1 and false discovery rate (FDR) < 0.05 using the limma package. Next, we used the univariate Cox regression model to analyze the prognosis of DEGs, with *p* < 0.01 as the standard. We then extracted the genes with significant prognostic significance for principal component analysis (PCA) and extracted principal component 1 as the signature score. Subsequently, all the patients were randomly assigned to a training set (1/2 for all patients) and a test set (1/2 for all patients). We used a similar method to define the immune score (IS) (Sotiriou et al., 2006; Zeng et al., 2019; Zhang et al., 2020).


ImmuneScore=ΣPC1-iΣPC1j

where *i* is the expression of DEGs whose Cox coefficient is positive, and *j* is the expression of DEGs whose Cox coefficient is negative.

### Predicting the Response to Immunotherapy

The immunophenoscore (IPS) is a quantitative score for tumor immunogenicity and is divided into 0–10 points. The IPS predicts the patient’s response to ICI treatment, and the IPS value is positively correlated with tumor immunogenicity (Charoentong et al., 2017). The IPS data were downloaded from The Cancer Immunome Atlas^2^.

### Statistical Analysis

R language software (Version 4.0.1) was used for statistical analysis. The Wilcox *T*-test was used to compare variables between groups. Univariate Cox regression analysis was used to assess the relationship between the total survival time and expression value of DEGs from the ccRCC cohort. With a *p*-value < 0.01 as the screening criteria, the prognostic value of this gene was considered statistically significant. The predictive accuracy of the immune score model was assessed by time-dependent receiver operating characteristic (ROC) curves using the survival ROC package. A *p*-value < 0.05 was considered statistically significant if no specific explanation was available.

## Results

### Landscape of Immune Cell Infiltration in Clear Cell Renal Cell Carcinoma

**FIGURE 1:**
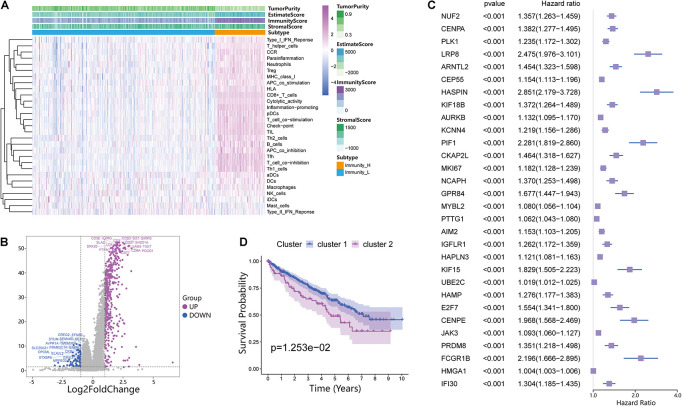
Investigation of the immune infiltration-dependent expression change in clear cell renal cell carcinoma (ccRCC). **(A)** Twenty-nine immune-related gene sets were enriched in ccRCC. These genes comprised immune cells and immune processes. The tumor purity, estimates, immunity scores, and stromal scores are also shown in the heatmap. Immunity High: Immunity_H, Immunity Low: Immunity_L. **(B)** Volcano plot of 514 genes differentially expressed between Immunity_L and Immunity_H. Purple dots and blue dots represent upregulated and downregulated genes, respectively. The screening criteria were | log2FC| > 1.0 and *p*-value < 0.05. **(C)** Univariate Cox regression analysis was used to screen genes significantly associated with prognosis with a *p*-value < 0.01. The top 30 genes are shown in the forest map. **(D)** Survival analysis of Cluster 1 and Cluster 2. In Cluster 1 and Cluster 2, the Kaplan–Meier curve with a log-rank *p*-value of 0.013 showed significant survival differences.

The 530 ccRCC samples in the TCGA database were scored by ssGSEA to quantify the activity, enrichment level and function of immune cells in each sample, and then they were divided into two groups using hierarchical cluster analysis (Figure 1A and Supplementary Figure 1A). Next, we used ESTIMATE to evaluate the level of immune cell infiltration, tumor purity, and matrix content (stromal score) of each ccRCC sample and defined the two clusters as Immunity High (Immunity_H) and Immunity Low (Immunity_L) (Figure 1A). We found that the stromal score and immune cell infiltration were significantly higher in the Immunity_H group than in the Immunity_L group, and tumor purity was significantly lower in the Immunity_H group group than in the Immunity_L group. To further investigate the reasons for the differences in immune activity between the different groups, we analyzed the gene expression changes between the Immunity_H and Immunity_L cohorts. We obtained a total of 437 upregulated genes and 77 downregulated genes in the Immunity_H cohort using | log2FC| > 1 and FDR < 0.05 as the criteria (Figure 1B). To further obtain DEGs related to prognosis, 514 DEGs were subjected to univariate Cox regression analysis. The genes were reserved for subsequent unsupervised cluster analysis with a *p*-value < 0.01. According to the screening criteria, 182 DEGs related to prognosis were obtained (Supplementary File 1), and the top 30 are shown in Figure 1C. To specifically investigate the role of these candidate DEGs in different immune subgroups, we divided the ccRCC samples into different subgroups according to the expression similarity of these related genes using the ConsensusClusterPlus package in R language (Supplementary Figures 1B–F). A *k* value of 2 proved to be the most suitable choice for dividing the ccRCC patient cohort into two clusters—namely, Cluster 1 and Cluster 2 (Supplementary File 2). Survival analysis showed that the two subtypes had obvious clinical significance, and the prognosis of Cluster 2 was significantly worse than that of Cluster 1 (Figure 1D). Therefore, we believed that these 182 DEGs could be used to assess the immune status of each patient with ccRCC and to predict the prognosis of the patients.

### Generation of Immune-Related Gene Signatures and Functional Annotation

**FIGURE 2:**
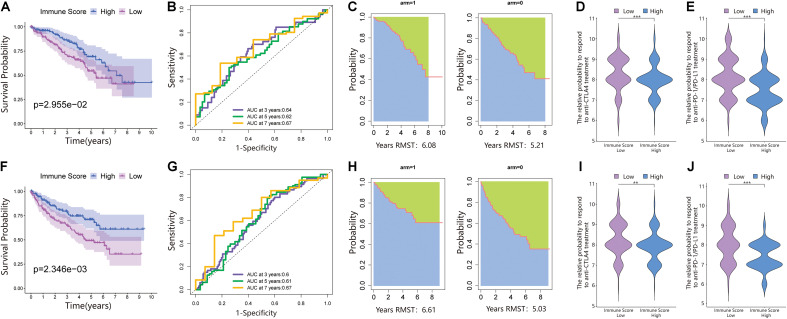
An immune score was constructed in the training set and verified in the validation set. **(A)** Survival analysis of the ISL group and ISH group in the training set. In these two groups, the Kaplan–Meier curve with a log-rank *p*-value of 0.030 showed significant survival differences. **(B)** Time-dependent receiver operating characteristic (ROC) curve analysis of the immune score in the training set. The 3-, 5-, and 7-year area under curves (AUCs) in all samples were 0.64, 0.62, and 0.67, respectively. **(C)** The restricted mean survival (RMS) curve for the immune scores was plotted in the training set. In the ISL and ISH groups, the RSM times were 6.08 and 5.21, respectively. The blue part represents the RMS time, and the green part represents the restricted mean time lost (RMTL). **(D,E)** The relative probabilities of anti-cytotoxic T lymphocyte-associated protein 4 (CTLA4) and anti-programmed cell death protein 1 (PD-1)/programmed death-ligand 1 (PD-L1) treatment between the immune score low (ISL) and ISH groups in the training set. **(F)** Survival analysis of the ISL group and ISH group in the testing set. In these two groups, the Kaplan–Meier curve with a log-rank *p*-value of 0.002 showed significant survival differences. **(G)** Time-dependent ROC curve analysis of the immune score in the testing set. The 3-, 5-, and 7-year AUCs in all samples were 0.60, 0.61, and 0.67, respectively. **(H)** The RMS curve for immune scores was plotted in the testing set. In the ISL and ISH groups, the RSM times were 6.61 and 5.03, respectively. The blue part represents the RMS time, and the green part represents the restricted mean time lost (RMSL). **(I,J)** The relative probabilities of anti-CTLA4 and anti-PD-1/PD-L1 treatment between the ISL and ISH groups in the testing set.

We performed PCA on the gene expression matrix of 530 ccRCC samples, extracted principal component 1 of 182 DEGs, and constructed the immune score (IS). Subsequently, all patients were randomly assigned to a training set (1/2 for all patients) and a testing set (1/2 for all patients). According to the IS, the samples were defined as the immune score low (ISL) group and immune score high (ISH) group in the training set, testing set, and total set. In the training set, compared with the ISH group, the overall survival of the ISL group was significantly reduced (Figure 2A). For overall survival (OS) prediction, the 3-, 5-, and 7-year AUCs of the ROC curve were 0.64, 0.62, and 0.67, respectively, which were higher than 0.6 and had good survival prediction ability (Figure 2B). The survRM2 package was used to calculate the restricted mean survival time (RMS time) of ccRCC patients during the 9-year follow-up. The RMS time is simply the overall average of the event-free survival time during the initial period of follow-up. This period can be evaluated by calculating the area under the KM curve. The RMS time in the ISH group was 6.08 years, and that in the ISL group was 5.21 years, a finding that also confirmed a better prognosis in the high group (Figure 2C). The predictive ability of the immune score was further verified in the testing set and total set.

According to the immune score constructed above, each patient in the test set and total dataset was divided into the ISL group and ISH group. Survival analysis showed that patients with high immune scores had longer OS in the testing set (Figure 2F). The 3-, 5-, and 7-year AUCs of the ROC curve were 0.6, 0.61, and 0.67, respectively (Figure 2G). The RMS time in the ISH group was 6.61 years, and that in the ISL group was 5.03 years (Figure 2H).

**FIGURE 3:**
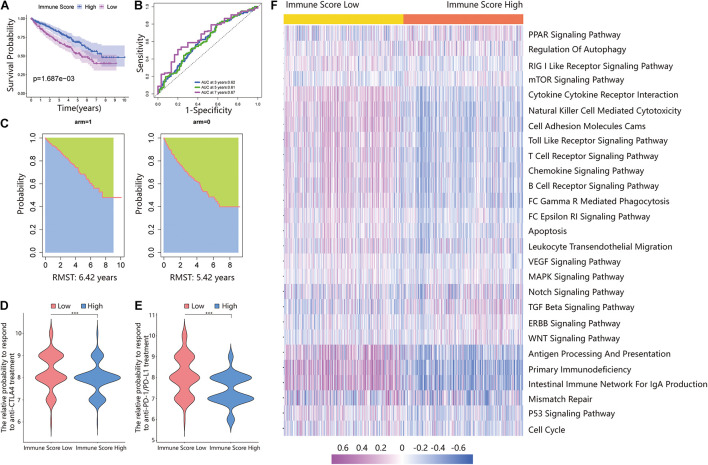
Validating the immune score in the total dataset. **(A)** Survival analysis of the ISL group and ISH group in the total set. In these two groups, the Kaplan–Meier curve with a log-rank *p*-value of 0.002 showed significant survival differences. **(B)** Time-dependent ROC curve analysis of the immune score in all samples. The 3-, 5-, and 7-year AUCs in all samples were 0.62, 0.61, and 0.67, respectively. **(C)** The RMS curve for the immune scores was plotted for all ccRCC samples. In the ISL and ISH groups, the RSM times were 6.42 and 5.42, respectively. The blue part represents the RMS time, and the green part represents the restricted mean time lost (RMSL). **(D,E)** Relative probabilities of anti-CTLA4 and anti-PD-1/PD-L1 treatment between the ISL and ISH groups in the total set. **(F)** Gene set variation analysis (GSVA) enrichment analysis of the activation states of biological pathways in distinct immune score groups. These biological processes are shown in the heatmap. Purple represents the activated pathway, and blue represents the inhibited pathway.

Additionally, the prognosis of the ISL group in the total dataset was significantly worse than that of the ISH group (Figure 3A). The 3-, 5-, and 7-year AUCs of the ROC curve were 0.62, 0.61, and 0.67, respectively (Figure 3B). The RMS time in the ISH group was 6.42 years and that in the ISL group was 5.42 years (Figure 3C).

### The Immune Score and Response of Patients to ICI Treatment

Because of the lack of immunotherapy response data matching patients in the TCGA database, we used the IPS value to replace the patient’s immunotherapy response. We extracted two IPS values (IPS-PD-1/PD-L1/PD-L2_pos and IPS-CTLA-4_pos) from the TCIA database to measure the response of ccRCC patients to anti-PD-1/PD-L1 and anti-CTLA4 treatment alternatives. The ISL group had a higher relative probability of responding to anti-PD-1/PD-L1 and anti-CTLA4 treatment in the training set, testing set, and total set (Figures 2D,E,I,J, 3D,E). These results indicated that patients with low immune scores were more likely to benefit from immunotherapy.

### Functional Annotation and Pathway Enrichment of the Immune Score

**FIGURE 4:**
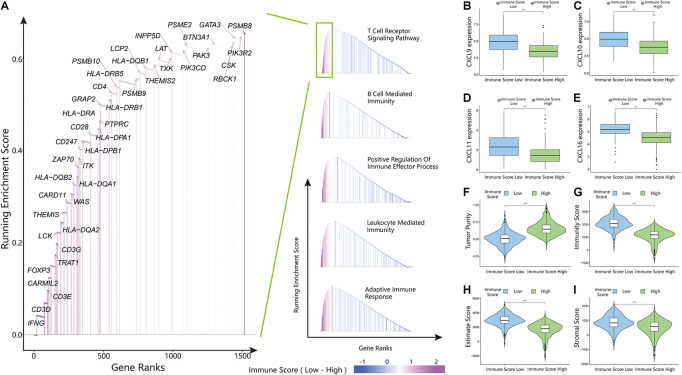
Gene set enrichment analysis (GSEA), chemokine expression, and estimateScore results in the ISL and ISH groups. **(A)** Gene ontology (GO) enrichment analysis of the activation states of immune-related pathways in distinct immune score groups. **(B–E)** Expression of CXCL9/10/11/16 between the ISL and ISH groups. The upper and lower ends of the box indicate the interquartile range of values. The line in the box indicates the median value, and the black dot indicates the outlier. The asterisk indicates the statistical *p*-value (***<0.001). **(F–I)** Violin plot of the estimateScore between the ISL and ISH groups, including the tumor purity **(F)**, immunity score **(G)**, estimate score **(H)** and stromal score **(I)**. The asterisk indicates the statistical *p*-value (***<0.001).

The above results proved the accuracy of the immune score model. Therefore, we used the total set for subsequent analysis. To further explore the biological behaviors among different immune groups, we performed GSVA enrichment analysis for KEGG pathway analysis in the total set. The ISL group was markedly enriched in immune-related pathways, such as natural killer cell-mediated cytotoxicity, the T-cell receptor signaling pathway, the B-cell receptor signaling pathway, and primary immunodeficiency (Figure 3F). The enrichment pathways in the ISH group were mainly concentrated in the TGF beta signaling pathway, PPAR signaling pathway, and WNT signaling pathway (Figure 3F). Similarly, we used GSEA to perform GO analysis to reveal specific biological processes related to immunity. The biological processes in the ISL group were mainly related to the T-cell receptor signaling pathway, B-cell-mediated immunity, positive regulation of the immune effector process, leukocyte-mediated immunity, and adaptive immune response (Figure 4A). Therefore, we believed that the constructed immune score could determine the immune status of different groups and predict the prognosis. Interestingly, we found that the immune status was active in the ISL group, but the prognosis of patients was worse in the ISL group. From the above, we hypothesized that although the immune state was active in the ISL group, its function might be inhibited.

### Immune Cell Infiltration in Clear Cell Renal Cell Carcinoma

**FIGURE 5:**
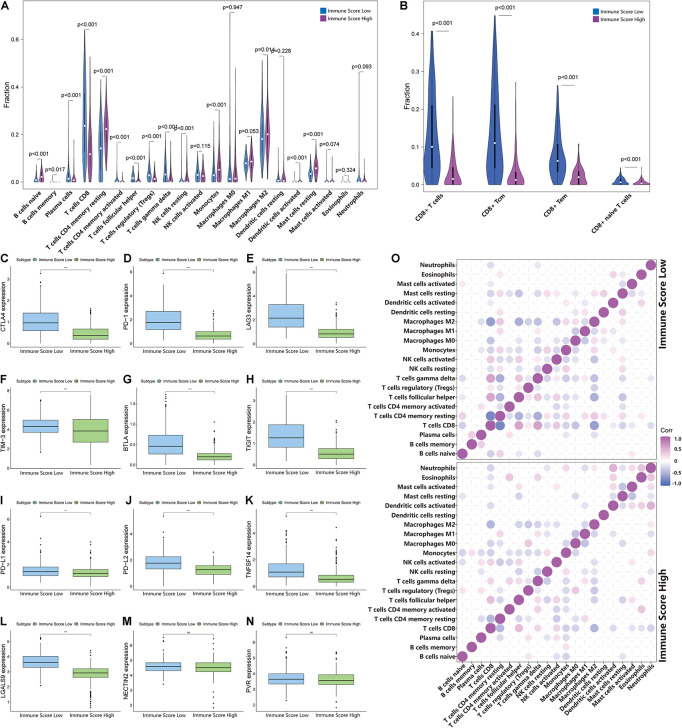
Changes in immune cell infiltration in different immune score groups. **(A,B)** The horizontal axis and vertical axis represent tumor infiltrating immune cells and relative percentages, respectively. Blue and purple represent the ISL group and ISH group, respectively. The data were evaluated using Wilcox tests. **(C–N)** The expression of inhibitory receptors and inhibitory ligands of CD8^+^ T cells between the ISL group and ISH group. The upper and lower ends of the box indicate the interquartile range of values. The line in the box indicates the median value, and the black dot indicates the outlier. Asterisks indicate statistical *p*-values (**<0.01, ***<0.001). **(O)** The relationship between the abundance ratios of different tumor immune infiltrating cells. The dot indicates that the *p*-value is less than 0.05, and the area is negatively correlated with the *p*-value. Purple indicates a positive correlation, and blue indicates a negative correlation.

To identify the infiltration status of immune cells in different groups, CIBERSORT was used to process the data and select samples with *p*-values less than 0.05. The immune infiltration landscapes of the ISL group (*n* = 253) and ISH group (*n* = 166) are shown in Supplementary Figure 2A, and the correlations of immune cells are shown in Figure 5O and Supplementary File 3. Different immune cells were weakly correlated or moderately correlated in tumor tissues in both subgroups. The interaction between immune cells was higher in the lower group than in the higher group. The highest positive correlations were found with CD8^+^ T cells and gamma delta T cells, follicular helper T cells, and activated NK cells in the ISL group. The highest negative correlations were found with CD8^+^ T cells and resting memory CD4^+^ T cells, and M2 macrophages in ISL. In the ISH group, only follicular helper T cells had a higher positive correlation. Similarly, resting memory CD4 T cells and M2 macrophages had a negative correlation with CD8^+^ T cells. In the ISL group, the degree of infiltration of CD8^+^ T cells, gamma delta T cells, regulatory T cells (Tregs), and follicular helper T cells were significantly higher than that in the ISH group (*p* < 0.05) (Figure 5A). Likewise, the degree of infiltration of resting memory CD4^+^ T cells, activated dendritic cells, M2 macrophages, and monocytes was higher in the ISH group. To accurately distinguish the status of CD8^+^ T cells, we used xCell to specifically quantify their classification (Supplementary File 4; Aran et al., 2017). The infiltration degree of CD8^+^ central memory T cells (Tcm) and CD8^+^ effective memory T cells (Tem) in the ISL group was higher than that in the ISH group (Figure 5B). Tem had a rapid effector function and easily differentiated into effector T cells, which secreted a large amount of IFN and was highly cytotoxic. Tcm also differentiated into effector T cells, but the differentiation speed was slower than that of Tem. At the same time, analysis of HLA expression in the two groups also confirmed the difference in the immune infiltration status (Supplementary Figure 2B). Additionally, the TIME correlation scores of the two groups showed significant differences (Figures 4F–I). This finding was consistent with our previous results that the infiltration and activation of CD8^+^ T cells in the ISL group were higher than those in the ISH group. Many CD8^+^ memory T cells in the low immune group were also potential targets for immunotherapy.

### Factors That Regulate the Recruitment and Activation of CD8^+^ T Cells

The above results indicated that the degree of infiltration of CD8^+^ T cells in the ISL group was higher than that in the ISH group, but the degree of activation was lower than that in the ISH group. By comparing the chemokines of CD8^+^ T cells, we found that the expression levels of *CXCL9/10/11/16* in the ISL group were significantly higher than those in the ISH group (*p* < 0.001) (Figures 4B–E). These results indicated that other components of the tumor microenvironment in the ISL group secreted these chemokines to recruit more CD8^+^ T cells into the tumor tissue. At the same time, we explored the expression of inhibitory receptors and ligands in CD8^+^ T cells. In the ISL group, the inhibitory receptors of CD8^+^ T cells, such as *CTLA4*, *PD-1*, *LAG3*, *TIM-3*, *BTLA*, and *TIGIT*, were significantly increased compared with those in the ISH group (*p* < 0.001) (Figures 5C–H). In addition to *NECTIN2* and *PVR*, the inhibitory ligands *PD-L1*, *PD-L2*, *TNFSF14*, and *LGALS9* of CD8^+^ T cells were also significantly increased in the ISL group (*p* < 0.01) (Figures 5I–N). From the above results, although the infiltration degree of CD8^+^ T cells increased in the ISL group, their functions were significantly inhibited. This finding might explain why the prognosis of the ISL group was worse than that of the ISH group.

### Comparisons of Somatic Mutations Under Different Immune Score Groups

**FIGURE 6:**
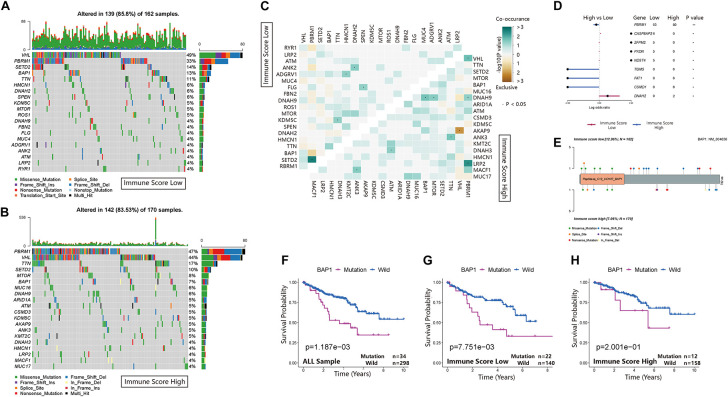
Landscape of somatic mutations in the ISL and ISH groups. **(A,B)** Waterfall plot of tumor somatic mutations established by those with low immune scores (A) and high immune scores **(B)**. Each column represents an individual patient. The bar plot above the figure shows the tumor mutation burden (TMB), and the number on the right represents the mutation frequency of each gene. **(C)** The heat map shows the mutual co-occurring and exclusive mutations of the top 20 frequently mutated genes. The color or symbol of each cell represents the statistical significance of the exclusivity or co-occurrence of each pair of genes, respectively. Green represents mutual co-occurrence, and brown represents exclusive mutation. Asterisks indicate statistical *p*-values (^⋅^<0.05). **(D)** Forest plot of statistically significant mutant genes between the groups. Asterisks indicate statistical *p*-values (*<0.05, **<0.01) **(E)** The lollipop plot illustrates the differential distribution of variants for BRCA1-associated protein 1 (BAP1). **(F–H)** Kaplan–Meier curves show the independent relevance between the overall survival time and BAP1 mutation in the ISL group, ISH group and all cohorts.

The waterfall map showed the highly mutated genes and their mutation classifications in the ISL (*n* = 162) group and ISH group (*n* = 170) (Figures 6A,B). In the ISL group, 139 patients had somatic mutations altered, accounting for 85.8%. In the ISH group, 142 patients had somatic mutations, accounting for 83.53%. In the ISL group, the top five genes with mutation frequencies were *VHL*, *PBRM1*, *SETD2*, *BAP1*, and *TTN*. In the ISH group, the top five genes with mutation frequencies were *PBRM1*, *VHL*, *TTN*, *SETD2*, and *MTOR*. The most common types of mutations were missense mutations in both the ISL and ISH groups (Supplementary Figures 3A,B). In the ISL and ISH groups, the median value of variants was 42 and 40, respectively, with no significant difference. Single nucleotide polymorphisms (SNPs) were the most common type of variation compared with deletions (DELs) and insertions (INSs). Additionally, C > T had the highest incidence among the six variation types in both groups. Interestingly, *VHL* and *PBRM1* both had a higher mutation frequency in ccRCC. However, no significant difference was found after comparing the mutation sites in the two cohorts (Supplementary Figures 3C,F). This finding indicated that they might have a lower effect on the infiltration of immune cells in tumor tissues and were more involved in tumorigenesis. At the same time, we used the maftools package to obtain drug-gene interactions and druggability information. Supplementary Figures 3D,E show the potential gene categories for drug therapy and the top five genes involved in them. Subsequently, we investigated co-occurring and exclusive mutations in the top 20 most frequently mutated genes (Figure 6C). In the two cohorts, *PBRM1*-*SETD2* and *PBRM1*-*LRP2* showed significant co-occurrence. This finding indicated that they might have redundant effects in the same pathway, and they had selective advantages between them that could retain multiple mutant copies. Additionally, some of the genes had different mutation frequencies in the two groups. Fisher’s test was used to detect the differentially expressed genes with a *p*-value less than 0.05 (Figure 6D). We further analyzed the effect of these genes with higher mutation frequency on the survival prognosis in different cohorts and all sample cohorts. Except for *BAP1*, mutations in other genes had no significant effect on the prognosis in different cohorts (Figure 6E) or all sample cohorts (Figures 6F–H and Supplementary Figures 2C–V).

### BRCA1-Associated Protein 1 Mutation Pattern in the Immune Score Cohort of Clear Cell Renal Cell Carcinoma

**FIGURE 7:**
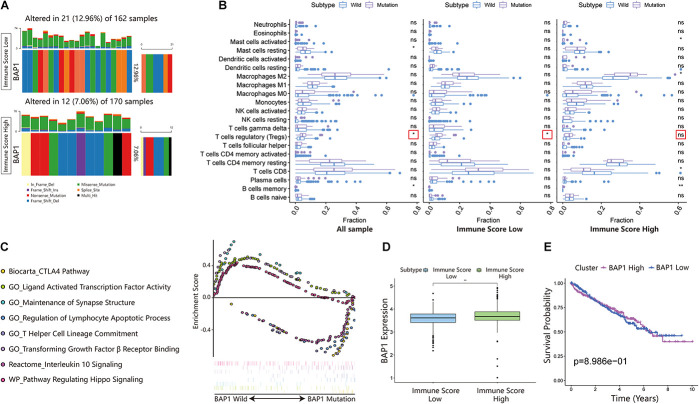
Changes in BAP1 mutations in ISL and ISH groups. **(A)** Mutation frequency of BAP1 in the ISL group and ISH group. Each column represents an individual patient. The bar plot above the figure shows the TMB, and the number on the right represents the mutation frequency of each gene. **(B)** Effect of BAP1 mutation on tumor immune infiltrating cells in the ISL group, ISH group, and whole samples (*p*-value, * < 0.05, ** < 0.01, ns > 0.05). **(C)** Gene set enrichment analysis comparing the BAP1 phenotype between the mutation group and wild-type group with FDR < 0.25. **(D)** The expression of BAP1 between the ISL group and ISH group (*p*-value, ** < 0.01). **(E)** The survival curve shows that the expression level of BAP1 is not related to the prognosis of ccRCC patients.

BRCA1-associated protein 1 (BAP1) is a tumor suppressor that regulates multiple processes, such as cell cycle control, programmed cell death, DNA damage repair, chromatin modification, and the immune response. In the ISL group, the mutation frequency of *BAP1* was 12.96% higher than that of the ISH group (7.06%) (Figure 7A). Additionally, by analyzing the infiltration of immune cells in different immune score cohorts and whole sample groups, we found that *BAP1* mutation might regulate the immune response in tumor tissues by affecting Treg cells (Figure 7B). At the same time, we used GSEA to analyze the biological behavior difference between the *BAP1* mutant and *BAP1* wild type. The *BAP1* mutation was mainly enriched in the CTLA4 pathway, T helper cell lineage commitment, interleukin 10 signaling and regulation of lymphocyte apoptotic process, while wild-type *BAP1* was mainly enriched in ligand-activated transcription factor activity, maintenance of synapse structure, pathway regulating Hippo signaling and transforming growth factor β receptor binding (Figure 7C). Additionally, the expression level of *BAP1* in the ISL group was significantly lower than that in the ISH group (*p* < 0.01) (Figure 7D). Furthermore, the prognosis of patients with high *BAP1* expression was similar to that of patients with low expression (Figure 7E). These results indicated that the *BAP1* mutation could regulate the immune response in tumor tissues.

## Discussion

With the increased use of immunotherapy, many studies have investigated potential immunotherapy markers. Presently, the choice of immunotherapy is mainly based on the level of expression of immune checkpoints, leading to some patients not benefiting from immunotherapy. Thus, in a complex tumor environment, it is difficult for drugs to achieve a perfect therapeutic effect based only on the immune checkpoint. Our study aimed to screen and identify genes related to immune infiltration and tumor mutation in the tumor microenvironment and to accurately identify not only patients but also drugs suitable for immunotherapy. We propose new research ideas concerning immunotherapy for other tumors.

In the present study, we first used ssGSEA to quantify the activity, enrichment level, function, and pathway of immune cells in each sample. Next, through hierarchical cluster analysis, the patients were divided into an immunity high group and an immunity low group. We identified differentially expressed genes by comparing the two groups. We believed that these DEGs were immune-related DEGs. Subsequently, univariate Cox regression analysis was used to further screen the prognosis-related DEGs. Based on these genes, the patients were divided into two groups (Cluster 1 and Cluster 2) through unsupervised cluster analysis. The prognosis of Cluster 2 was worse than that of Cluster 1. Therefore, we speculated that the prognosis-related DEGs could be used as genes to construct immune score models. After that, we used principal component analysis to extract principal component 1 and calculated the immune score according to relevant literature (Sotiriou et al., 2006; Zeng et al., 2019; Zhang et al., 2020). All the patients were randomly assigned to a training set (1/2 for all patients) and a test set (1/2 for all patients). We used the training set to construct the immune score model and verified it in the validation set and total set. The patients were divided into ISL and ISH groups according to the immune score. The 3-, 5-, and 7-year AUCs were all greater than 0.6, and the RSM time in the ISH group was longer than that in the ISL group, proving the sensitivity and predictability of the immune score model. Thus, the immune score model accurately predicted the prognosis of patients. Therefore, we used the total set for subsequent analysis. Using GSEA and GSVA, differences were found in immune-related pathways between the groups. By comparing HLA-related genes and related scores in the immune microenvironment, we also confirmed that the immune activity status of the ISL group was higher than that of the ISH group. Interestingly, the ISL group, which had a poor prognosis, had a higher level of immune activity. Hence, we speculated whether the function of immune cells was inhibited in the ISL group. We used CIBERSORT and xCell to further quantify the types of infiltrating immune cells in each sample. CD8^+^ T cells are the main immune killer cells in tumor tissues. Therefore, we evaluated the status of CD8^+^ T cells in different immune score groups. Because of the increased secretion of *CXCL9/10/11/16* chemokines, we believed that the infiltration of CD8^+^ T cells in the ISL group was significantly higher than that in the ISH group. At the same time, the inhibitory ligands and inhibitory receptors of CD8^+^ T cells in the ISL group were also significantly increased compared with those in the ISH group. Therefore, we hypothesized that despite the higher immune activity and infiltration of CD8^+^ T cells in the ISL group, smart tumor cells would express their inhibitory ligands to prevent its function. This finding may also explain the poor prognosis in the ISL group. In the low group, the massive infiltration of Tem and Tcm cells also serve as potential targets for subsequent immunotherapy. Although the model has certain deficiencies in the accuracy of the prognosis and effect of immunotherapy, we recognize that the reason may be the insufficient number of modeling samples. If conditions permit continued expansion of the sample size, the accuracy of the model may be further improved. Additionally, if the sequencing data before and after immunotherapy can be further collected, further optimization of the model will be valuable.

Generally, two indicators are related to tumor immunotherapy: the degree of immune cell infiltration and tumor mutation burden. Therefore, to accurately select patients suitable for immunotherapy, both factors must be considered. By comparing the mutant landscapes of the ISL group and ISH group, we found that the mutation frequency of the two groups was not significantly different. Both *VHL* and *PBRM1* had very high mutation rates in both groups. This finding confirmed that *VHL* and *PBRM1* played a key role in the pathogenesis of ccRCC (Gu et al., 2017; Hsieh et al., 2017; Zhang J. et al., 2018; Cai et al., 2019). However, the genes with a high mutation frequency did not significantly affect the prognosis compared with all the other genes except *BAP1*, whether in the ISL group, ISH group or whole sample group. *BAP1* regulates DNA damage repair pathways (Nishikawa et al., 2009; Zhao et al., 2017), the cell cycle, and cell proliferation (Yu et al., 2010; Okino et al., 2015), chromatin (Scheuermann et al., 2010), and cell death pathways (Sime et al., 2018; Zhang Y. et al., 2018). Additionally, *BAP1* is implicated in immune regulation (Gezgin et al., 2017; Figueiredo et al., 2020). However, the mechanism of IMMUNE regulation by *BAP1* remains unclear. The mutation frequency of *BAP1* in the ISL group was higher than that in the ISH group. The effects of *BAP1* mutation on infiltrating immune cells were compared in the ISL group, ISH group, and full sample group. *BAP1* mutation increased the infiltration degree of Treg cells. The *BAP1* mutation was mainly enriched in the CTLA4 pathway, T helper cell lineage commitment, interleukin 10 signaling, and regulation of the lymphocyte apoptotic process pathway by GSEA. These results indicated that *BAP1* mutation can inhibit the activity of immune cells in tumor tissues by regulating Treg cells. Therefore, we believe that combining the immune score and *BAP1* mutation can better screen patients suitable for immunotherapy. Additionally, inhibitors of Treg cells can be combined to achieve better therapeutic effects.

## Conclusion

Our study predicted the prognosis of renal cancer patients by constructing a new immune score combined with the *BAP1* mutation. At the same time, it provides a new way to identify patients suitable for immunotherapy and explore effective immunotherapy strategies in ccRCC patients.

## Data Availability Statement

The datasets presented in this study can be found in online repositories. The names of the repository/repositories and accession number(s) can be found in the article/Supplementary Material.

## Author Contributions

HH and KL designed the study and analyzed the data. ZG and JC wrote the manuscript and performed the data analysis. YT and SY critically revised the draft for important intellectual content. JZ and QW participated in the picture drawing and processing. SP and JC performed the statistical analysis. ZX was mainly responsible for article modification and technical support. All authors read and approved the final manuscript.

## Conflict of Interest

The authors declare that the research was conducted in the absence of any commercial or financial relationships that could be construed as a potential conflict of interest.

## Publisher’s Note

All claims expressed in this article are solely those of the authors and do not necessarily represent those of their affiliated organizations, or those of the publisher, the editors and the reviewers. Any product that may be evaluated in this article, or claim that may be made by its manufacturer, is not guaranteed or endorsed by the publisher.

## Footnotes

1
https://portal.gdc.cancer.gov/

2
https://tcia.at/

## Supplementary Material

Supplementary Figure 1 Unsupervised clustering grouping in ccRCC. **(A)** Unsupervised hierarchical clustering algorithm in ccRCC patients. **(B–F)** Unsupervised clustering of 182 DEGs in the ccRCC cohort and consensus matrices for *k* = 2–5.

Supplementary Figure 2 Immune infiltration status and prognosis of different mutated genes in different immune score groups. **(A)** Immune cell abundance ratios in the two immune scoring cohorts. Each column represents a sample, and each column uses a different color and height to indicate the abundance ratio of immune cells in the sample. **(B)** The RNA expression levels of HLA genes in samples from the ISL group and ISH group (*p*-value, ^∗∗∗^ < 0.001). **(C–V)** K-M analysis of genes with higher mutation frequencies in ccRCC samples and different immune score cohorts.

Supplementary Figure 3 Mutations in different immune score groups and possible drug pathways. **(A,B)** Summary of the mutation information with statistical calculations. The mutation types were classified according to different categories. Among them, missense mutations accounted for the largest proportion, SNPs appeared more frequently than insertions or deletions, and C > T was the most common mutation in SNVs. The top 10 mutated genes in the two cohorts are also displayed. **(C,F)** The lollipop plot illustrates the differential distribution of variants for VHL and PBRM1. **(D,E)** The potential gene categories for drug therapy in the two cohorts and the top five genes involved.

Supplementary File 1 The univariate Cox regression analysis results of DEGs with the *p*-value < 0.01.

Supplementary File 2 The groups of unsupervised cluster analysis.

Supplementary File 3 The correlations of immune cells in the ISL and ISH groups.

Supplementary File 4 The quantitative results of immune infiltrating cells in tissues of ccRCC patients used by xCell.
