# Supplementary figures and images for: Immune Signatures Combined With BRCA1-Associated Protein 1 Mutations Predict Prognosis and Immunotherapy Efficacy in Clear Cell Renal Cell Carcinoma
Source: Front Cell Dev Biol. 2021 Oct 18;9:747985. doi: 10.3389/fcell.2021.747985 (PMC8558467; doi:10.3389/fcell.2021.747985)

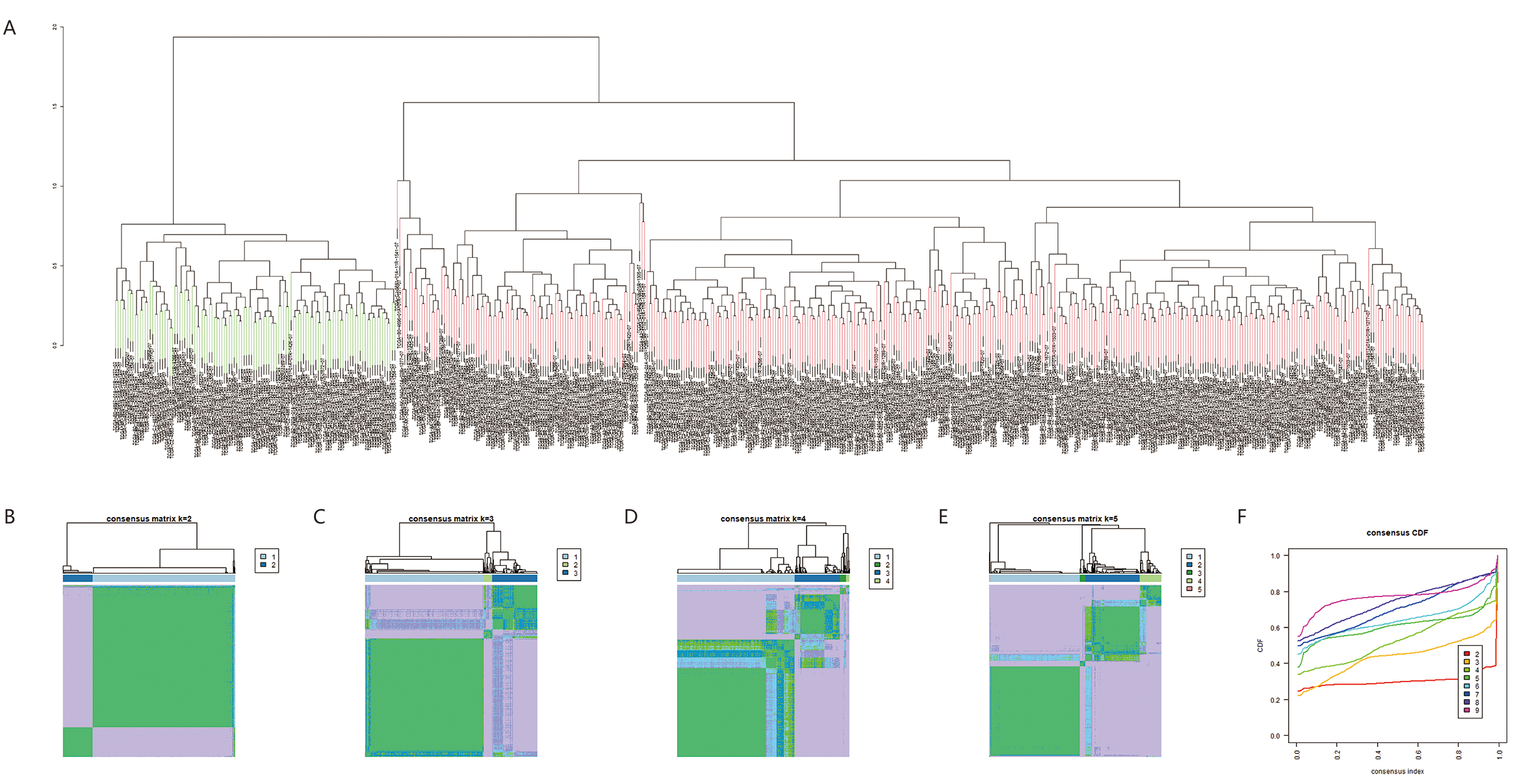

Supplement: Supplementary Figure 1 — Unsupervised clustering grouping in ccRCC. (A) Unsupervised hierarchical clustering algorithm in ccRCC patients. (B–F) Unsupervised clustering of 182 DEGs in the ccRCC cohort and consensus matrices for k = 2–5. [file Image_1.TIF]

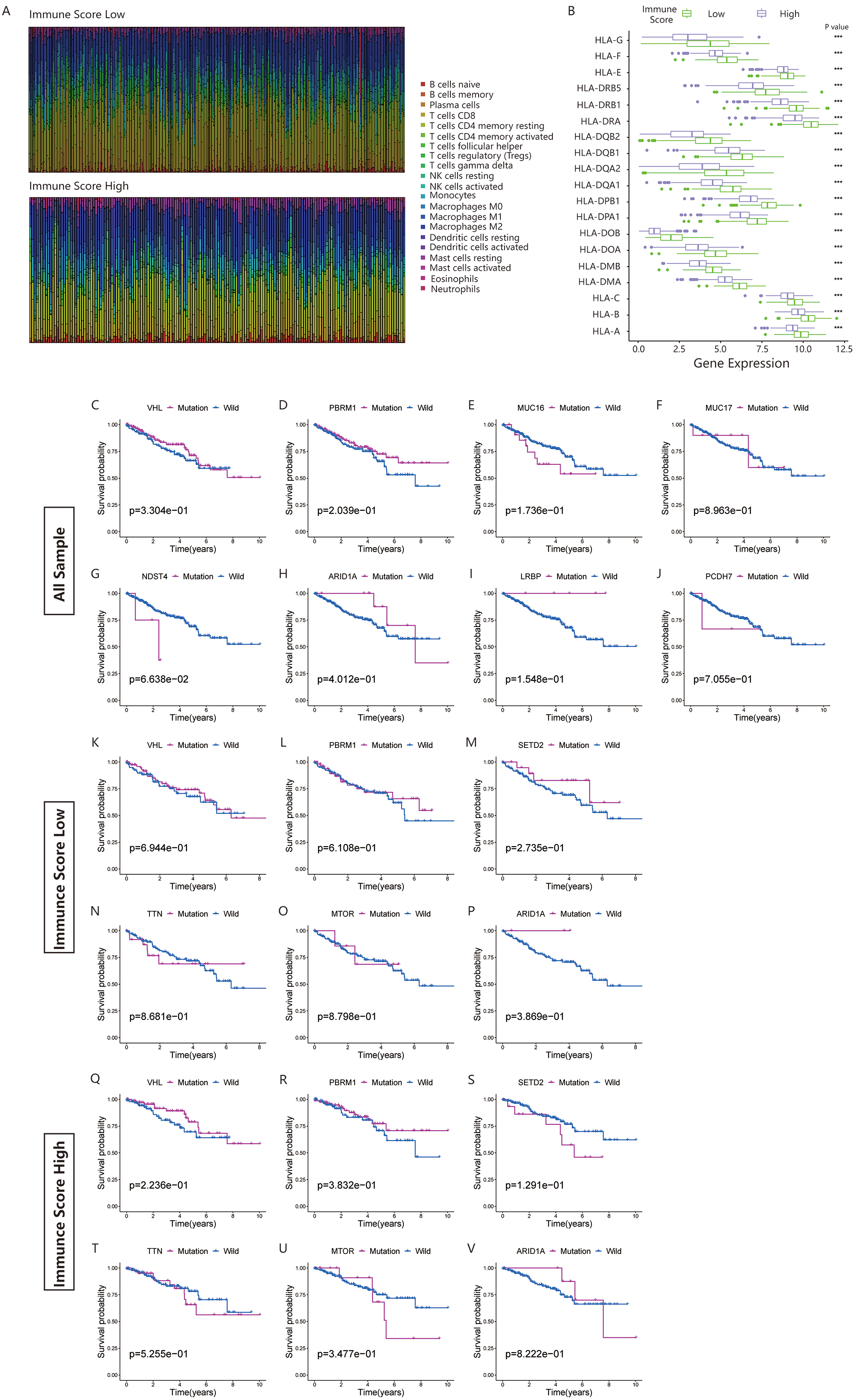

Supplement: Supplementary Figure 2 — Immune infiltration status and prognosis of different mutated genes in different immune score groups. (A) Immune cell abundance ratios in the two immune scoring cohorts. Each column represents a sample, and each column uses a different color and height to indicate the abundance ratio of immune cells in the sample. (B) The RNA expression levels of HLA genes in samples from the ISL group and ISH group (p-value, ∗∗∗ < 0.001). (C–V) K-M analysis of genes with higher mutation frequencies in ccRCC samples and different immune score cohorts. [file Image_2.TIF]

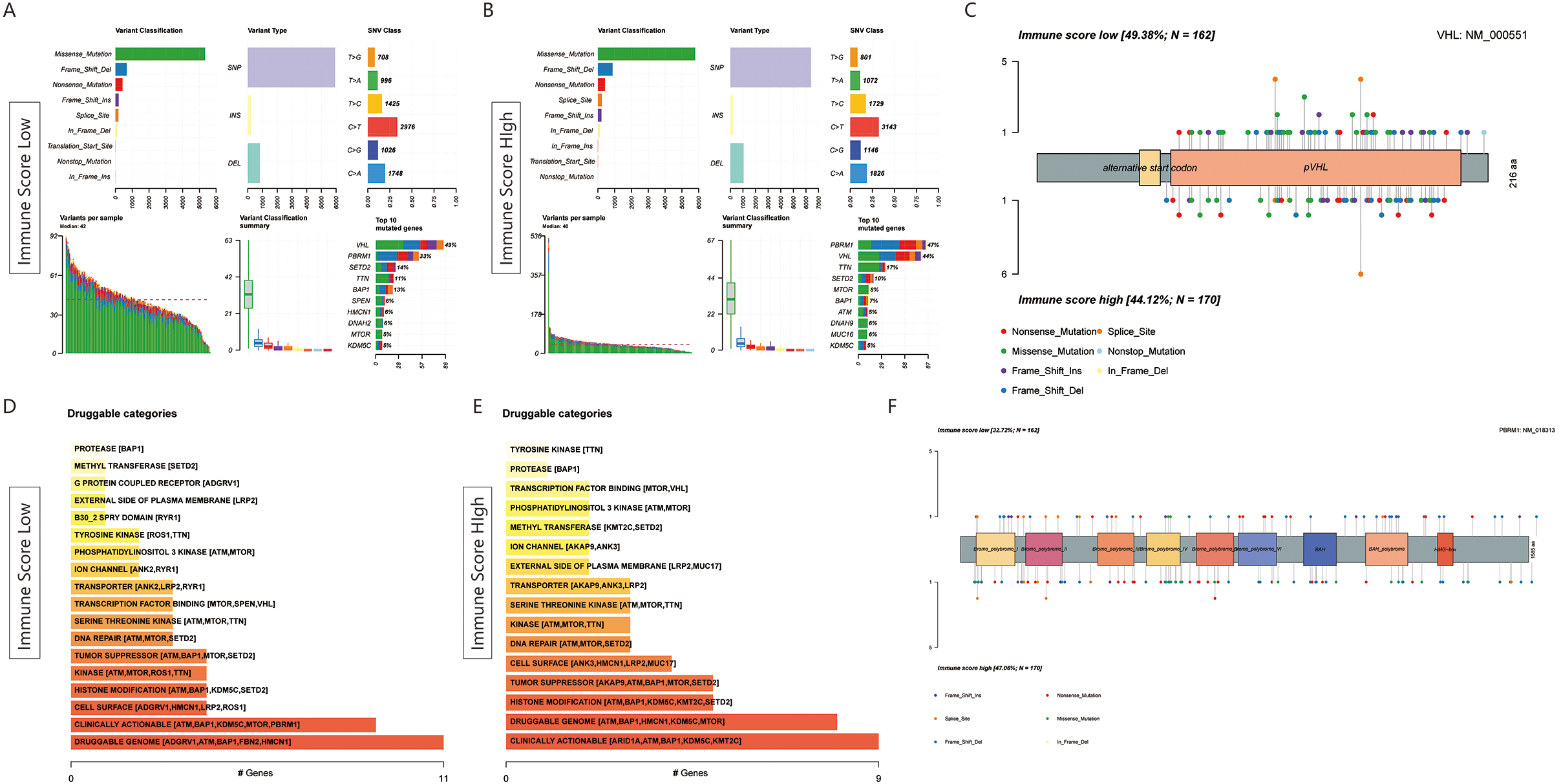

Supplement: Supplementary Figure 3 — Mutations in different immune score groups and possible drug pathways. (A,B) Summary of the mutation information with statistical calculations. The mutation types were classified according to different categories. Among them, missense mutations accounted for the largest proportion, SNPs appeared more frequently than insertions or deletions, and C > T was the most common mutation in SNVs. The top 10 mutated genes in the two cohorts are also displayed. (C,F) The lollipop plot illustrates the differential distribution of variants for VHL and PBRM1. (D,E) The potential gene categories for drug therapy in the two cohorts and the top five genes involved. [file Image_3.TIF]
